# Mothers as advocates for healthier lifestyle behaviour environments for their children: results from INFANT 3.5-year follow-up

**DOI:** 10.1186/s12889-022-14659-8

**Published:** 2022-11-29

**Authors:** Christine Delisle Nyström, Karen J Campbell, David Crawford, Kylie D Hesketh

**Affiliations:** 1 Department of Biosciences and Nutrition, Karolinska Institutet, Neo, 141 83 Huddinge, Sweden; 2 Institute for Physical Activity and Nutrition (IPAN), Deakin University, 3125 Geelong, Australia

**Keywords:** Advocacy, Lifestyle behaviours, Pre-school children

## Abstract

**Background:**

The amount of time children spend outside of their home environment has increased over the past decades. Therefore, the quality of the health behaviour environments where young children spend time is likely to impact their health behaviour opportunities. The aim of this study was to describe the proportion of mothers who consider it possible to make changes in their local communities to increase opportunities for children to eat healthily, be physically active, and limit screen time exposure, as well as the proportion who have attempted to do so. The characteristics of mothers with differing advocacy beliefs and intentions were explored.

**Methods:**

Cross-sectional data collected using questionnaires from 307 mothers who participated in the 3.5-year follow-up of the INFANT cluster-randomized controlled trial were used. Frequencies were used to assess the number of mothers who thought it possible to bring about change in their local communities and for the those who had attempted to do so. Binary logistic regression analyses were used to compare sociodemographic characteristics of mothers with differing responses.

**Results:**

Most mothers thought it was possible to bring about change in their local community with regards to providing more opportunities for their child to eat healthily (83.7%), be physically active (90.9%) as well as limit exposure to screen time (63.5%). However, less than 19% and 11% of mothers have thought about or tried to bring about change in their child’s childcare centre or local community, respectively. No sociodemographic differences were found between the mothers who thought it was possible to bring about actioning change (*p*-values > 0.1) or for those that have thought about change (*p*-values > 0.1).

**Conclusion:**

As children are continuously being exposed to obesogenic environments future quantitative and qualitative studies are needed to describe how to promote parental advocacy and engagement, in order to provide children with environments that support healthy lifestyle behaviours.

**Supplementary Information:**

The online version contains supplementary material available at 10.1186/s12889-022-14659-8.

## Background

In 2020 approximately 39 million children under five years of age were classified as overweight or obese [1]. Unhealthy lifestyle behaviours such as a poor diet, inadequate physical activity, and excessive sedentary behaviour are well recognized individual determinants of childhood obesity [2]. Furthermore, obesity-related behaviours have been found to be established in childhood and track into adulthood [3, 4]. Thus, there is a great need to promote healthy lifestyle behaviours in key environments where young children spend time: in the home, childcare, and community environments.

The amount of time spent in childcare has increased over recent decades due to increased employment rates of mothers [5], which has led to the childcare centre becoming an important setting for the promotion of healthy diet and physical activity behaviours [6]. In Australia there are a range of childcare services available (e.g., long day care, family day care, pre-school, and informal care), with approximately two-thirds of one to four year old children attending some type of childcare [7]. According to the National Quality Framework, childcare in Australia should promote and support every child’s physical activity and health and ensure that for every child healthy eating and physical activity are appropriate and promoted, but specific benchmarks are not set [8]. Depending on the type of care, children either bring their own meals and snacks or they can be provided by the childcare provider [9, 10]. On the website for the Australian Dietary Guidelines it specifies that discretionary foods and beverages should not be included at childcare [11]; however, jurisdictional differences for discretionary food recommendations have been observed in Australia, ranging from some never having these foods on the regular menu to some allowing them up to once per day [9]. Furthermore, with regards to physical activity there is a lack of written policies specifying the amount of physical activity children should participate in while in childcare [12]. Thus, given the broad wording with regards to quality standards in childcare, coupled with the lack of written policy for physical activity, screen time, and diet, individual centres have much autonomy around their environment, thus there may be opportunity for parents to influence these factors within childcare.

The community environment, such as the local playgrounds and parks as well as food stores and vendors, can also influence physical activity and diet through having appropriate play equipment for a variety of age groups and through food advertising and availability. While parents play a key role in creating and supporting healthy lifestyle behaviours in the home environment [13], with more time being spent outside of the home environment parents need to ensure that childcare centres and community environments are concurrently promoting healthy lifestyle behaviours for their children.

The Melbourne Infant Feeding Activity and Nutrition Trial (INFANT) was an obesity prevention intervention targeting Australian parents. The intervention was initiated when infants were four months of age and was 15 months in duration [14]. Long-term follow-ups of these families were then conducted at 2- and 3.5-years post-intervention [15]. Long-term follow-ups in intervention studies provide ideal opportunities to collect data outside the trial’s scope that is of interest to this population, which will hopefully promote future research. At the 3.5-year follow-up in INFANT questions regarding parental advocacy for promoting healthier diet and physical activity environments were added to the parental questionnaire as this is an area that is currently understudied.

Therefore, the aim of this study is to describe the proportion of mothers who consider it possible to make changes in their local communities to increase opportunities for children to eat healthily, be physically active and limit screen time exposure, as well as the proportion who have attempted to do so. We also explored the characteristics of mothers who believe it is possible to bring about change, as well as those who have thought about advocating for change in their communities regarding their child’s diet, physical activity, and screen time.

## Methods

### Study design and participants

INFANT was a community-based cluster randomized controlled trial. The overall aim of this trial was to prevent obesity through the promotion of healthy lifestyle behaviours (i.e., promote positive diet and physical activity and reduce sedentary behaviours). Details regarding the study design, sample selection, and intervention have previously been described in detail [14, 16]. Briefly, the control-arm received usual care from maternal and child health services, whereas the intervention-arm received usual care plus the INFANT program (comprised of six group sessions complemented with take-home material over 15 months). The intervention content focused on dietary intake, sedentary behaviour, active play, as well as the family food, physical activity, and sedentary behaviour environments. Outcomes were assessed at baseline, post-intervention (15 months after baseline), and at two long-term follow-ups (2- and 3.5-years post-intervention). This study was approved by the Deakin University Human Research Ethics Committee (2007 − 175) and conducted in accordance with the Declaration of Helsinki.

At baseline, 542 children were randomized into INFANT with 492 families completing post-intervention measurements. All families that completed the trial were asked to participate in the long-term follow-ups [15]. This cross-sectional study utilizes data from the 307 mothers that had complete data for the three questions regarding parental advocacy in their local community in the main caregiver’s questionnaire (i.e., Do you think it is possible for parents to bring about change in their local community to provide more opportunities for children to eat healthily (yes or no) or to be physically active (yes or no); and to limit children’s exposure to screen time (yes or no)). Based on this inclusion criteria there were no statistically significant differences between the mothers included or excluded based on the aforementioned questions who participated in the 3.5-year follow-up with regards to age, body mass index (BMI), or educational attainment (all *p*-values > 0.05).

### Measures

#### Mothers as advocates of health

In the main caregiver’s questionnaire at the 3.5-year follow-up (child age approximately five years old), mothers were first asked the following three questions: whether they think it is possible for parents to bring about change in their local community to provide more opportunities for children: (1) to eat healthily?; (2) to be physically active?; and (3) to limit children’s exposure to screen time? (yes or no). The aforementioned questions were created taking the transtheoretical model of behaviour change into account [17].

Subsequently, they were asked whether they had thought about, and separately whether they had tried to bring about, change in their child’s childcare centre, their child’s kindergarten/preschool/school, or elsewhere in their local community for each of the aforementioned behaviours (18 questions in total). For each question there were three answer options (yes, no, and not applicable). For this manuscript the categories child’s childcare centre and child’s kindergarten/preschool/school were combined into one category called childcare centre.

#### Maternal sociodemographic characteristics

At the time of study enrolment all mothers completed a demographic questionnaire regarding their age, weight, and height (self-reported), as well as educational attainment, which was dichotomized into two categories: high school/trade school or university in order to maximize power.

#### Maternal concern

At the 3.5-year follow-up concern was assessed in the main caregiver’s questionnaire where mothers were asked to indicate their level of concern regarding: (1) their child’s weight; (2) their child’s diet; (3) their child’s physical activity level; (4) their child’s television viewing; and (5) their child’s use of computers and electronic games, each on a five-point Likert scale [18].

#### Statistical analyses

Frequencies were used to assess the number of mothers who thought it possible to bring about change in their local communities. For those who had answered `yes´ to at least one of the questions relating to the three health behaviours (*n* = 280), we then calculated the number and frequencies of the parents who had thought or tried to bring about change in their child’s childcare centre or elsewhere in their local community with regards to providing more opportunities for children to eat healthily and be physically active or limit exposure to screens. A dichotomous composite score variable was created (i.e., yes or no/not applicable) indicating whether parents have thought about bringing about change in any setting for diet, physical activity, or screen time (if they answered `yes´ to any of the nine questions they were categorized as `yes´).

Binary logistic regression analyses accounting for the cluster-based recruitment (i.e., by parent group in order to adjust for parents in the same community) were used to assess potential differences in maternal sociodemographic characteristics (n range = 306–307) and maternal concern (*n* = 307) between mothers who had answered `yes´ to at least one of the three questions regarding beliefs that it was possible to bring about change in their local community versus `no´ to all. Binary logistic regression analyses were also used to assess the cross-sectional associations between the composite score indicating whether mothers had thought about bringing about change with maternal sociodemographic characteristics (n range = 279–280) and maternal concern (*n* = 280). As there were no statistically significant differences between the intervention and control group regarding mothers who thought it was possible to bring about change, the data for both groups were pooled. All models were adjusted for intervention status. Stata (Release 15, StataCorp LP) was used for all analyses using a 5% level of significance.

## Results

The 307 children were on average 5.0 ± 0.1 years old and 165 (53.8%) were boys. Mothers were on average 37.3 ± 4.1 years old and 188 (61.2%) had a university education.

The majority of mothers thought it was possible to bring about change in their local community, with 83.7% (*n* = 257), 90.9% (*n* = 279), and 63.5% (*n* = 195) believing it is possible to advocate for opportunities to eat healthily, be physically active, and limit exposure to screen time, respectively. There were no statistically significant associations found for maternal age, educational attainment, pre-pregnancy BMI or concern with child behaviours between mothers who thought it was possible to bring about change in their local community compared to those who did not (all *p*-values > 0.1). Supplemental Table 1 provides the odds ratios and 95% confidence intervals for all associations (see Additional file 1).

Tables 1 and 2 display the number of parents who have thought or tried to bring about change in their child’s childcare centre or elsewhere in their local community, respectively. More parents had thought or tried to bring about change for diet compared to physical activity or screen time in both their child’s childcare centre and elsewhere in the local community. Furthermore, more parents had thought about bringing about change than had tried to do so. In fact, very few parents had tried to bring about change in their child’s childcare centre or elsewhere in the local community for any behaviour.

**Table 1:** Frequency of parents who have thought to bring about change (n, %) (*n* = 280)

	Childcare centre	Elsewhere in the local community
*Provide more opportunities to eat healthily* ^a^		
Yes	51, 18.3%	36, 13.1%
No	204, 73.4%	209, 75.7%
Not applicable	23, 8.3%	31, 11.2%
*Provide more opportunities to be physically active* ^b^		
Yes	24, 8.6%	31, 11.1%
No	239, 85.3%	224, 80.3%
Not applicable	17, 6.1%	24, 8.6%
*Limit exposure to screens* ^b^		
Yes	9, 3.2%	15, 5.4%
No	210, 75.0%	221, 79.2%
Not applicable	61, 21.8%	43, 15.4%

^a^Two and four parents had missing values thus *n* = 278 (childcare centre) and *n* = 276 (elsewhere in the local community)

^b^One parent had missing values thus *n* = 279 (elsewhere in the local community)

**Table 2:** Frequency of parents who have tried to bring about change (n, %) (*n* = 280)

	Childcare centre	Elsewhere in the local community
*Provide more opportunities to eat healthily* ^a^		
Yes	29, 10.4%	22, 7.9%
No	217, 77.8%	215, 77.6%
Not applicable	33, 11.8%	40, 14.5%
*Provide more opportunities to be physically active* ^b^		
Yes	16, 5.7%	17, 6.1%
No	229, 82.1%	221, 79.5%
Not applicable	34, 12.2%	40, 14.4%
*Limit exposure to screens*		
Yes	5, 1.8%	11, 3.9%
No	201, 71.8%	212, 75.7%
Not applicable	74, 26.4%	57, 20.4%

^a^One and three parent(s) had missing values thus *n* = 279 (childcare centre) and *n* = 277 (elsewhere in the local community)

^b^One and two parent(s) had missing values thus *n* = 279 (childcare centre) and *n* = 278 (elsewhere in the local community)

No statistically significant associations were observed between maternal concern, maternal age, educational attainment or BMI between those who had thought about bringing about change for any behaviour compared to those that have not (all *p*-values > 0.1). Supplemental Table 2 provides the odds ratios and 95% confidence intervals for all associations (see Additional file 1). These comparisons could not be made for parents who tried to bring about change given the very low prevalence.

## Discussion

Parents are often considered natural advocates for their child, owing to the long-term obligation to their child’s well-being [19]. Throughout the past few decades, the most common parental advocates are those that have children with special needs [19–21], and little research has been conducted in other populations or settings. In the present study, the majority of mothers thought it was possible to bring about change in their local community with regards to providing more opportunities to eat healthily, to be physically active as well as limit exposure to screen time. However, only about one fifth of mothers had in fact thought about or tried to bring about change in their child’s childcare centre and only around one tenth in their local community. Finally, we observed no sociodemographic differences or differences in the level of maternal concern between the mothers who thought it was possible to bring about change or for those that have thought to bring about change.

Qualitative studies with child healthcare workers have emphasised the need for support and good communication with parents to promote physical activity and a healthy diet in childcare settings [22–24]. However, other qualitative studies with child healthcare workers have proposed numerous barriers to parental involvement. These include that childcare workers perceive parents as too busy to engage in conversation and when time did permit topics other than nutrition were prioritized [25] or they were not concerned about the promotion of physical activity [26]. The Centers for Disease Control and Prevention created `Parents for Healthy Schools´, which is a resource for parents on how they can engage to promote healthy schools and it includes both nutrition and physical activity components [27]. In the facilitators guide they highlight the importance of educating school staff in order to develop their abilities to improve parent engagement in school health [28]. This suggests that in order to engage parents in creating healthier school or childcare environments, the idea probably needs to be initiated from school or childcare staff and then parents can take the next step and create parent teacher associations or wellness committees.

Today’s society is increasingly obesogenic with food environments [29] and the built environment [30] being associated with childhood obesity. A recent study in Australia found that almost 75% of outdoor food advertisements within 500 m of primary and secondary schools promoted unhealthy foods and beverages [31]. This is a significant concern as exposure to food advertising has been found to be associated with an increase in food intake in children [32]. With regards to the built environment, a recent systematic review including 55 studies in 2 to 6-year-old children found that in the school domain that greater availability of fixed playground equipment, open spaces, and a large variety of portable play equipment had a positive impact on children’s physical activity levels [33]. This clearly demonstrates that there are areas where parents can advocate for change to create healthier local environments for their children; however, the question remains on how to promote parental engagement.

According to a policy brief from UNICEF regarding marketing of unhealthy foods and beverages to children, there is a need to form allied groups, as unified voices have greater chances to raise awareness and create change [34]. Furthermore, according to McCammon et al. [19] service providers can aid parents to grow as advocates through informing as well as providing training and other opportunities. In Australia, Cancer Council Victoria created the campaign `Food Fight!´, which brings together parents/families, experts, and organizations to stop the marketing of unhealthy foods and beverages where children play, learn, and commute [35]. Thus, there is potential through educational/advocacy campaigns like `Food Fight!´, for parents to learn and become empowered, which could possibly lead to the formation of parental advocacy groups in the local communities who advocate to childcare centres, school boards and local governments to create better food and built environments for children.

Finally, the number of mothers who thought about or tried to bring about change to limit their child’s exposure to screens in childcare or in the local community was much lower in comparison to those that have thought about or tried to bring about change to provide more opportunities to eat healthy and be more physically active. In the home environment parents of young children have found it difficult to regulate screen use [36], which may be a reason why less mothers have reported thinking about or have tried to limit screen time outside the home environment in the present study. However, it is also possible that mothers in the current study did not perceive screen time in childcare as an issue, since screens are not often available in this setting in Australia. Furthermore, in the local community (e.g., parks and playgrounds) are a setting which often encourages physical activity and mothers in this study may not perceive screen time as a problem in this environment. More research, both quantitative and qualitative is needed to further investigate parental perceptions regarding advocating for healthier screen time environments for their children.

To the best of our knowledge this is the first quantitative study investigating parents as advocates for change for healthier lifestyle behaviours in both childcare and community environments. Further strengths of this study are the relatively large sample size of parents, questions regarding advocacy across a range of behaviours, as well as questions spanning from pre-contemplation to action. This study is limited by its cross-sectional design with the questions regarding advocacy only asked at the 3.5-year follow-up as well as the fact that the questions have not been validated. Additionally, the intervention and control group were pooled together for these analyses which is another potential limitation. However, it is important to highlight that INFANT did not discuss or promote parental advocacy and there were no differences between groups with regards to the number of mothers who thought it was possible to bring about change for the three health behaviours. Additionally, the fixed answer questions asked in the present study are limited, as we were not able to get the underling rationale of the mothers’ answers. Thus, future studies should include both fixed and open-ended questions to help better understand parents’ opinions and reasoning with regards to advocating for healthier lifestyle behaviours in both childcare and the local community.

## Conclusion

The majority of mothers in INFANT reported that they thought it was possible to bring about change in their local community with regards to providing more opportunities to eat healthily, to be physically active as well as limit exposure to screen time. As children are continuously being exposed to obesogenic environments future quantitative and qualitative studies are needed to describe how to promote parental advocacy and engagement, in order to provide children with environments that support healthy lifestyle behaviours.

## Supplementary Information

**Additional file 1: Supplemental Table 1.** Associations between maternal demographic characteristics and concern with mothers who thought it was possible to bring about change in their local community. **Supplemental Table 2.** Associations between maternal demographic characteristics and concern with mothers who have thought to bring about change in their child’s childcare centre or elsewhere in the local community.

## Data Availability

The datasets are available upon reasonable request to the corresponding author.

## Abbreviations

BMI: Body mass index
INFANT: The Melbourne Infant Feeding Activity and Nutrition Trial
