# Supplementary material for: Mothers as advocates for healthier lifestyle behaviour environments for their children: results from INFANT 3.5-year follow-up
Source: BMC Public Health. 2022 Nov 29;22:2211. doi: 10.1186/s12889-022-14659-8 (PMC9706958; doi:10.1186/s12889-022-14659-8)
Supplement: Supplementary file 1 — Additional file 1: Supplemental Table 1. Associations between maternal demographic characteristics and concern with mothers who thought it was possible to bring about change in their local community. Supplemental Table 2. Associations between maternal demographic characteristics and concern with mothers who have thought to bring about change in their child’s childcare centre or elsewhere in the local community. [file 12889_2022_14659_MOESM1_ESM.docx]

**Supplemental table 1.** Associations between maternal demographic characteristics and concern with mothers who thought it was possible to bring about change in their local community.

|  | **n** | **Odds ratio** | **95% CI** | **p-value** |
| --- | --- | --- | --- | --- |
| *Maternal characteristics* |  |  |  |  |
| Age | 307 | -0.06 | -0.17, 0.05 | 0.31 |
| Educational attainment | 307 | 0.33 | -0.44, 1.11 | 0.40 |
| Pre-pregnancy BMI | 306 | -0.05 | -0.12, 0.02 | 0.16 |
| *Maternal concern about* |  |  |  |  |
| Child’s weight | 307 | -0.57 | -1.42, 0.27 | 0.18 |
| Child’s diet | 307 | 0.37 | -0.29, 1.03 | 0.27 |
| Child’s physical activity | 307 | 0.40 | -0.68, 1.49 | 0.47 |
| Child’s tv viewing | 307 | -0.66 | -1.46, 0.14 | 0.11 |
| Child’s use of computers/games | 307 | -0.29 | -1.07, 0.49 | 0.47 |

Abbreviations: CI, Confidence interval; BMI, Body mass index

**Supplemental table 2.** Associations between maternal demographic characteristics and concern with mothers who have thought to bring about change in their child’s childcare centre or elsewhere in the local community.

|  | **n** | **Odds ratio** | **95% CI** | **p-value** |
| --- | --- | --- | --- | --- |
| *Maternal characteristics* |  |  |  |  |
| Age | 280 | 0.02 | -0.03, 0.07 | 0.48 |
| Educational attainment | 280 | 0.31 | -0.15, 0.77 | 0.18 |
| Pre-pregnancy BMI | 279 | -0.02 | -0.07, 0.03 | 0.50 |
| *Maternal concern about* |  |  |  |  |
| Child’s weight | 280 | -0.36 | -1.07, 0.35 | 0.32 |
| Child’s diet | 280 | 0.01 | -0.47, 0.49 | 0.97 |
| Child’s physical activity | 280 | -0.11 | -0.78, 0.56 | 0.75 |
| Child’s tv viewing | 280 | 0.08 | -0.42, 0.58 | 0.75 |
| Child’s use of computers/games | 280 | 0.04 | -0.54, 0.62 | 0.89 |

Abbreviations: CI, Confidence interval; BMI, Body mass index
